# Advances in intestinal flora for the development, diagnosis and treatment of CRC

**DOI:** 10.3389/fmicb.2025.1495274

**Published:** 2025-05-29

**Authors:** Ruiyao Hu, Yuting Qiu, Dong-ang Liu, Shiyu Chen, Keyi Chen, Yue Xu, Jinghua Yuan, Xinling Zhang, Xiaoping Li

**Affiliations:** Key Laboratory of Artificial Organs and Computational Medicine in Zhejiang Province, Shulan International Medical College, Zhejiang Shuren University, Hangzhou, China

**Keywords:** colorectal cancer, intestinal flora, pathogenesis, screening, prevention and treatment

## Abstract

Colorectal cancer (CRC), being prevalent among digestive tract malignancies, exhibits substantial mortality and morbidity rates. The intestinal microbiota, predominantly located in the colorectum, is diverse and comprises both conditionally pathogenic bacteria that can promote CRC development and probiotics that can inhibit it to some extent. Intestinal flora is associated with colorectal cancer, affecting its onset and progression through metabolites, immune regulation, and damage to the intestinal mucosal barrier. The intestinal flora exhibits significant potential in the diagnosis and treatment of CRC. Certain bacterial species can serve as biomarkers for CRC, aiding in the detection of precancerous and early-stage lesions. For instance, alterations in the abundance of *Fusobacterium nucleatum (Fn)* and *Enterotoxigenic Bacteroides fragilis (ETBF)* may indicate an elevated risk of CRC. On the other hand, probiotics such as Bifidobacteria could modulate chemotherapy and immunotherapy, improving treatment outcomes and reducing side effects, making them an effective approach to prevent CRC etiology and act as an adjuvant therapy. This paper focuses on a review of the relationship between intestinal flora and CRC, sorting out its potential role in developing, diagnosing, and treating CRC. It will advance precise, intelligent, and individualised prevention and treatment for CRC.

## Graphical abstract

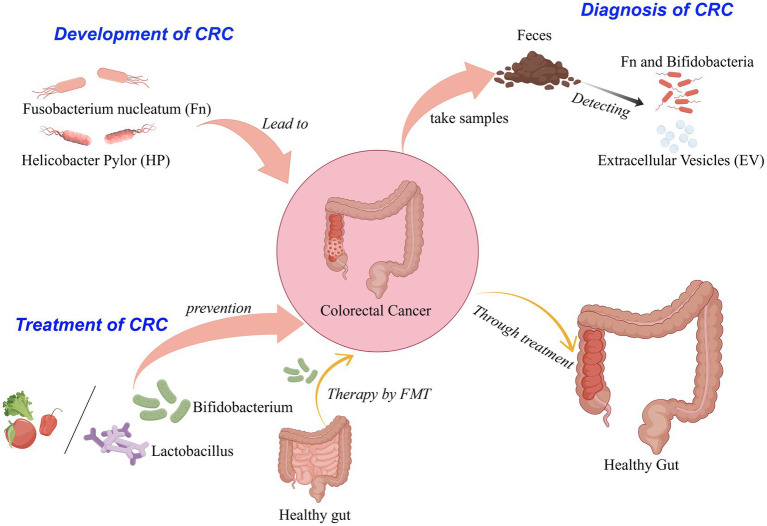

## 1 Introduction

Colorectal cancer (CRC), a prevalent malignancy affecting the digestive tract, stands as the third most frequently diagnosed cancer globally, comprising approximately 10 percent of total cancer occurrences. Furthermore, it ranks second as a primary contributor to cancer-related mortality, its seriousness is self-evident (World Health Organization, 2023). Early-stage CRC often lacks distinct symptoms; however, as the disease advances, it is commonly associated with various clinical manifestations, such as abdominal pain, an abdominal mass, hematochezia, intestinal obstruction, anemia, etc. At the same time, systemic symptoms such as emaciation, fatigue, and low fever may also occur. The occurrence of CRC is closely related to physiological, genetic, behavioral habits, lifestyle, disease, and other factors, among which the role of intestinal flora is significant. CRC is often diagnosed late with limited treatment options (Dekker et al., 2019). Therefore, elucidating the pathogenesis and progression of CRC is paramount for the development of efficacious preventative and therapeutic strategies.

The human microbiome is thought to be a collection of microorganisms whose genes and products have settled on our bodies since birth and transferred vertically (de Vos et al., 2022). Intestinal flora is a community of microorganisms, mainly composed of bacteria, that inhabit the gut. This extensive and intricate microbial community plays a crucial role in the intestinal ecosystem. In the intestinal flora, the dominant bacterial species belong to four major phyla, including *Firmicutes, Bacteroidetes, Actinobacteria*, and *Proteobacteria* (Zhou et al., 2021; Zhang et al., 2019). Among them, *Fusobacterium nucleatum (Fn)* is a bacterium that mainly exists in the human oral cavity and is rarely found in the lower digestive tract of healthy individuals. *Fn* is enriched in human colorectal neoplasms, and its presence in tumors supports cancer progression through multiple pathways and the regulation of immune responses, which impacts tumor recurrence, metastasis, and patient prognosis (Zepeda-Rivera et al., 2024). Gut microbiota is considered to be one of the key factors in regulating the health of the host. It contributes to forming a key part of the tumor microenvironment, interacting with host colonic epithelial and immune cells by releasing metabolites, proteins, and macromolecules that influence CRC development (Wong and Yu, 2023). The intestinal microbiota colonizes the human intestine, contributing to the absorption of nutrients and impacting digestive function, immune response, and drug metabolism in the body. From infancy to old age, its diversity and stability evolve dynamically with changes in diet, lifestyle, geographical location, and antibiotic use. Only about 6.6% of microbial taxa have genetic stability, while up to 48.6% of the variation of microbial taxa can be attributed to the shared effect of co-living environment (Gacesa et al., 2022). This paper examines the role of intestinal flora in the occurrence and development of CRC and summarizes its applications in early screening, diagnosis, prevention, and treatment.

## 2 The mechanism of intestinal flora on the occurrence and development of CRC

The normal intestinal flora and the environment in which it lives together constitute the intestinal micro-ecosystem. Under normal conditions, the structure and quantity of intestinal flora remain stable, jointly maintaining the balance of the intestinal micro-ecosystem. However, abnormal proliferation or reduction of bacteria such as *Fn, Helicobacter pylori, enterotoxigenic Bacteroides fragilis*, and *butyricum* will disrupt intestinal flora homeostasis. According to relevant research, when the homeostasis of intestinal flora is disrupted, there are marked alterations in both the species diversity and abundance of the microbiota (Fassarella et al., 2021). Such changes profoundly affect the expression of functional genes and the activity of metabolism-related enzymes, as well as the production of various detrimental metabolic byproducts. These consequences contribute to intestinal metabolic disorders, which in turn provoke chronic inflammatory responses in the gastrointestinal tract. This chronic inflammation can result in immune cell exhaustion and immune evasion, ultimately facilitating tumor development (Yin et al., 2023).

### 2.1 The effect of *Fusobacterium nucleatum (Fn)* on the development of CRC

*Fn*, a Gram-negative obligate anaerobic bacterium, belongs to the genus Fusobacterium and is primarily colonized in the human oral cavity. *Fn* can enter the gastrointestinal tract from the mouth through swallowing and spread throughout the digestive tract. In more than 40% of patients with CRC, researchers detected the same *Fn* cell nuclei in CRC and saliva samples (Komiya et al., 2019). According to recent studies, *Fn* is considerably enriched in colorectal cancer tissues and influences several stages of the cancer’s progression. This includes the promotion of tumor cell proliferation, immune evasion, recurrence, and chemoresistance, through multiple mechanisms. These mechanisms involve the modulation of host cytokine levels, the promotion of angiogenesis, and the inhibition of the organism’s immune regulatory capabilities (Zheng et al., 2024).

#### 2.1.1 *Fn* promotes tumorigenesis and proliferation

**Figure 1:**
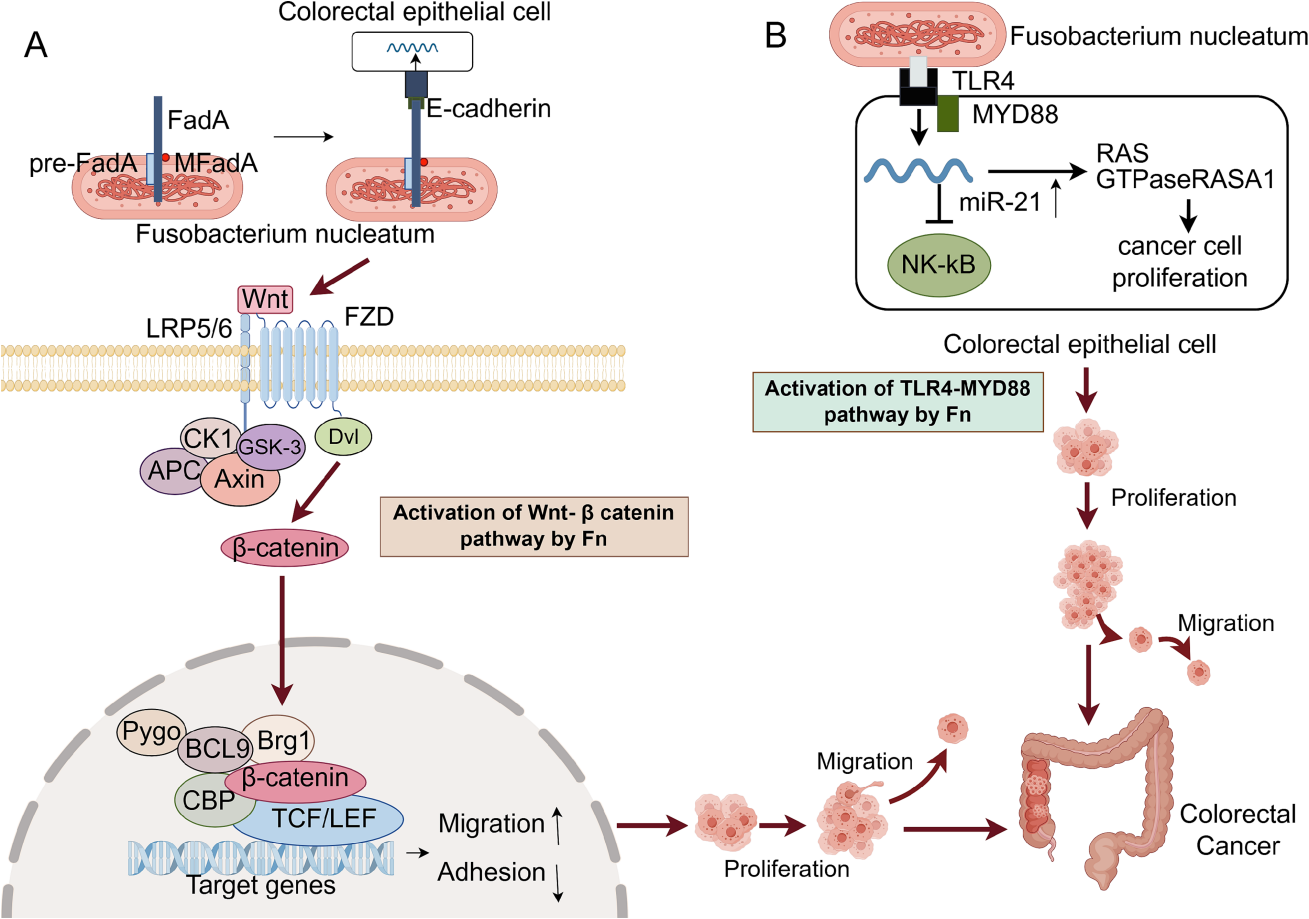
Two main mechanisms of *Fn* promoting CRC proliferation. CK1, Casein Kinase 1; GSK-3, Glycogen Synthase Kinase – 3; FZD, Frizzled receptors; Dvl, Dishevelled; APC, Adenomatous polyposis coli; CBP, CREB-binding protein; TLR4, Toll-like receptor 4. **(A)** FadA on the surface of *Fn* adheres to E-cadherin on the surface of host cells, triggering the Wnt / *β*-catenin signaling pathway. Wnt ligands bind to Frizzled receptors and form complexes with LRP5 / 6 on the cell surface to activate signal transduction. Subsequently, Dvl is phosphorylated and activated, leading to the inactivation of GSK-3, thereby preventing the degradation of β-catenin. β-catenin accumulates and transports into the nucleus, binds to the LEF / TCF transcription factor in the nucleus, activates the expression of Wnt target genes, and promotes the proliferation of CRC cells. **(B)** *Fn* activates the host cell’s TLR4 receptor through its LPS, initiating the NF-κB signaling pathway, which leads to an increase in the levels of miR-21. The upregulation of miR-21 suppresses the expression of RASA1, thereby relieving the suppression of the RAS signaling pathway and resulting in its activation. The activated RAS signaling pathway propels the cell cycle into the S phase, promotes DNA synthesis, and enhances the proliferation of CRC cells. This figure was drawn by Figdraw.

*Fn* promotes the process of tumorigenesis and proliferation, which mainly improves the proliferation ability of tumor cells through two different mechanisms. A FadA protein on the surface of *Fn* binds to E-cadherin in colorectal cancer cells, causing host-epithelial cell attachment, which then triggers the Wnt /*β*-catenin pathway (Wang and Fang, 2023). Wnt ligands, upon binding to LRP and Frizzled receptors, initiate a signaling cascade. This interaction activates a suite of protein complexes, including those comprising Axin, glycogen synthase kinase-3 (GSK-3), and casein kinase 1 (CK1). At the receptor, these complexes prevent the phosphorylation of β-catenin, which stops its degradation. Consequently, β-catenin gathers in the cytoplasm and is transported to the nucleus. In this context, it binds to transcription factors belonging to the T-cell factor/lymphoid enhancer factor (TCF/LEF) family, which in turn activates the downstream transcriptional program of the Wnt signaling pathway. The dysregulation of the Wnt pathway plays a major role in driving the growth, invasion, and survival of colon cancer cells (Bian et al., 2020). Concurrently, studies have demonstrated that this regulatory mechanism governs bacterial adhesion to and invasion of epithelial cells. It also modulates oncogene activity and increases their expression. Consequently, this up-regulation activates the expression of cyclin-dependent kinase 5 (Cdk5), thereby promoting the proliferation of colorectal cancer cells (Li et al., 2020). The other activates the RAS signaling pathway by activating the TLR4 receptor and NF-κB signaling pathway, leading to an increase in intracellular miR-21 and inhibiting the expression of RASA1, thereby inducing S-phase accumulation and enhancing the proliferation of CRC cells (Wang and Fang, 2023). In addition, after *Fn* invades host cells, it can produce amino acid metabolites such as methionine, leucine, and short-chain fatty acids (see Figure 1). These metabolites act as myeloid cell stimulators and can lead to myeloid cell expansion in tumors. Different *Fn* metabolites can directly promote tumor cell proliferation and vascular growth or inhibit the body’s immune effect on tumor cells, making the tumor microenvironment more prone to tumor growth over time (Li et al., 2022).

#### 2.1.2 *Fn* inhibits the immune response of the body

*Fn* inhibits the body’s immune response. The enrichment level of *Fn* in CRC tissues was negatively correlated with the number of tumor-infiltrating lymphocytes, resulting in anti-tumor immunosuppression (Wang and Fang, 2023; Hamada et al., 2018). *Fn* can promote the polarization of M2 macrophages through TRL4-dependent mechanisms and produce immunosuppressive effects, including activation of IL-6/ p-signal transducer and activator of transcription 3 (STAT3) / c-MYC pathway and NF-κB / S100 calcium-binding protein A9 (S100A9) pathway to protect the survival and proliferation of *Fn* in host cells (Chen et al., 2018; Hu et al., 2021). Fap2 functions as an adhesin, facilitating the recognition and adherence to colorectal cancer cells. Upon binding to TIGIT, a human inhibitory receptor is present on T lymphocytes and natural killer (NK) cells, and Fap2 initiates the TIGHT signaling pathway. This pathway suppresses the cytotoxic capabilities of these immune cells, consequently safeguarding *Fn* and adjacent tumor cells from immunological destruction. Moreover, *Fn* engages with and stimulates the human inhibitory receptor CEACAM1, thereby dampening the functional activity of T lymphocytes and NK cells (Gur et al., 2019). *Fn* may produce a local immunosuppressive effect by up-regulating the expression of TGFβ1 in colon cancer cells and mediating the immune escape of tumor cells.

#### 2.1.3 *Fn* promote the occurrence of inflammatory response

*Fn* promotes the occurrence of inflammatory response. Chronic colonic inflammation, or persistent host low-grade inflammation caused by intestinal microbiota stimulation, contributes to the occurrence and development of CRC. *Fn* can stimulate the upregulation of inflammatory and chemokine gene expression through multiple mechanisms, resulting in the release of inflammatory mediators and the recruitment of lymphocytes. This sets up an inflammatory environment that induces repeated local inflammation, causing cell damage and apoptosis, and could ultimately lead to CRC (Despins et al., 2021). Through the interaction between *Fn* Fap2 and host Gal-GalNAc, *Fn* can invade CRC cells and induce the secretion of IL-8 and CXCL1. These pro-inflammatory cytokines recruit adjacent immune cells and further promote the secretion of their cytokines to promote tumor inflammatory microenvironment (Casasanta et al., 2020).

### 2.2 The effect of *Helicobacter pylori (HP)* on the development of CRC

*HP* is a Gram-negative bacterium with curved, S-shaped, or spiral, flagella, strong activity, and no spores. It is the only microbial species known to survive in the human stomach. *HP* is linked not just to conditions such as gastritis, peptic ulcer disease, and gastric cancer, but it also appears to have a significant correlation with the genesis and progression of CRC. *HP* promotes CRC by altering the intestinal immune status, promoting inflammatory responses, aberrantly activating tumor signaling pathway, and disrupting the integrity of the intestinal barrier.

*HP* infection can competitively inhibit the proliferation of other bacteria in the gastrointestinal tract and cause an imbalance of microbial flora in the small and large intestines, but it does not change the overall microbial richness. Infection may lead to the increase of carcinogenic bacteria and mucus-degrading bacteria and the decrease of beneficial metabolites, and the toxic effects of itself and metabolites may promote tumor proliferation (Engelsberger et al., 2024; Ralser et al., 2023; Dash et al., 2019).

*HP* infection impairs the integrity of the intestinal barrier. The intestinal mucosal barrier is the key to maintaining homeostasis in the body. Intestinal mucosal barrier damage is an important trigger factor for intestinal flora to participate in the occurrence of colitis and CRC (Camilleri, 2019). The integrity of the intestinal barrier is dependent on mucus secretion by goblet cells, which support the intestinal lining. However, in contrast to the uninfected CRC mouse model, a reduced secretion of mucus was observed in both the small intestine and colon of wild-type and Apc-mutated mice following *HP* infection. This reduction suggests that *HP* may impair the intestinal barrier’s structural integrity (Ralser et al., 2023). In a murine model of chronic DSS-induced colitis, the CagA protein of *Helicobacter pylori* was found to compromise the integrity of the colonic mucosal barrier and facilitate the destruction of intestinal epithelial cells mediated by IFN-*γ* through a mechanism involving CagA-containing exosomes. Additionally, CagA upregulated the expression of Claudin-2 at the transcriptional level via a CDX2-dependent pathway, thereby impeding the recovery of damaged mucosa in an *in vitro* colitis model (Guo et al., 2022).

*HP* leads to abnormal activation of tumor signaling pathways. In related experiments, the gene expression of intestinal epithelial cells in mice infected with *HP* was found to be upregulated. Additionally, there was an observed aberrant activation of the STAT3 and NF-κB signaling pathways, which are associated with the initiation and progression of CRC. Activation of epithelial STAT3 is conducive to the recruitment of lymphocytes while inhibiting the infiltration of Treg cells in the colon, stimulating the abnormal proliferation and differentiation of CRA cells, leading to CRC (Ralser et al., 2023).

### 2.3 The effect of other intestinal flora on the development of CRC

There is a broad spectrum of normal flora in the gut, and the progression of colorectal cancer is intricately related to the intestinal flora, with different bacterial species influencing its development in different ways.

Some of the flora contribute to the development of CRC, among which *Enterotoxigenic Bacteroides fragilis (ETBF)* is a typical representative. *ETBF* produces the toxin BFT, which disrupts the colonic epithelial barrier by promoting the cleavage of the zonula adherens protein E-cadherin. This process initiates a cellular signaling response characterized by inflammation and c-myc-dependent oncogenic over-proliferation. Additionally, ETBF may enhance tumorigenesis by eliciting an IL-17-mediated carcinogenic inflammatory response through a Th17-mediated pathway, thereby facilitating the accumulation of regulatory T lymphocytes (Qu et al., 2023; Valguarnera and Wardenburg, 2020).

In addition, probiotics in the intestine, such as *Clostridium butyricum* and *Streptococcus thermophilus*, may help prevent CRC. *Clostridium butyricum* interacts with the Wnt/*β*-catenin signaling pathway, thereby inhibiting the proliferation of colorectal cancer cells and promoting apoptosis. This interaction also plays a role in regulating the composition of the intestinal flora (Chen et al., 2020). The production of β-Galactosidase by *Streptococcus thermophilus* increases the abundance of known probiotics in the intestine, and the production of galactose can regulate oxidative phosphorylation and Hippo signaling pathways, resulting in tumor inhibition (Qu et al., 2023; Li et al., 2021). In summary, the promotion or inhibition of different intestinal flora in CRC is briefly described in the Table 1.

**Table 1:** The relationship between common intestinal bacteria and CRC.

Intestinal flora	Gram stain	Effect in CRC	Pathogenesis	The interaction mechanism with other microorganisms	References
*Fn*	Negative	Promote	Promote the proliferation and migration of CRC cells Cdk5 up-regulation activates the Wnt / β-catenin signaling pathway and STAT3 pathways Activate TLR4 receptor and NF-κB signaling pathway, inhibit the expression of RASA1, activate RAS signaling pathway, induce s-phase accumulation, and enhance the proliferation of CRC cells Inhibition of immune response, inhibition of T cell activation and NK cell-induced killing of tumor cells Inflammation and chemokine expression fosters an inflammatory microenvironment, resulting in recurrent local inflammation, cell damage, and apoptosis.	None	Wang and Fang (2023), Li et al. (2020), Gur et al. (2019), and Despins et al. (2021)
*HP*	Negative	Promote	The toxic effects of itself and its metabolites may promote the proliferation of CRC cells Destroy the intestinal mucosal barrier and induce CRC Activation of the STAT3 pathway elevates inflammatory factors and gastrin levels, driving abnormal proliferation and differentiation in CRA cells	Promote carcinogenic bacteria	Engelsberger et al. (2024), Ralser et al. (2023), Dash et al. (2019), and Camilleri (2019)
*ETBF*	Negative	Promote	The accumulation of regulatory T cells to enhance the inflammatory response IL-17-mediated carcinogenic inflammatory response to increase tumorigenesis	Promote carcinogenic bacteria	Qu et al. (2023) and Valguarnera and Wardenburg (2020)
*Clostridium butylicum*	Positive	Inhibit	Interaction with Wnt / β-catenin signaling pathway Regulating the composition of intestinal flora Inhibit the proliferation of CRC cells and induce apoptosis	Intestinal flora regulation	Chen et al. (2020)
*Streptococcus thermophilus*	Positive	Inhibit	Producing β-Galactosidase to increase the abundance of intestinal probiotics Galactose production modulates oxidative phosphorylation and the Hippo signaling pathway, thereby inhibiting CRC	Intestinal flora regulation	Qu et al. (2023) and Li et al. (2021)

## 3 Application of intestinal flora in early screening and diagnosis of CRC

Since CRC is often found at a late stage with restricted treatment choices, early detection and diagnosis are critical. Because of the important role of intestinal flora in the occurrence and development of CRC, intestinal flora, and its related biomolecules are expected to be biomarkers for early screening and diagnosis of CRC.

At present, the predominant method for CRC detection is the Fecal Immunochemical Test (FIT). This test is characterized by its simplicity, convenience, and non-invasive nature, making it particularly suitable for large-scale population screening initiatives. Given that the intestinal microbiota of CRC patients exhibits significant differences compared to that of healthy individuals, specific microbial communities and their combinations may serve as valuable biomarkers for auxiliary diagnosis of CRC. Among these species, we observed an elevation of the enterotoxigenic bacterium *ETBF*, which is considered a key pathogen for CRC initiation. There was a significant increase in CRC-promoting species like *Fn, Parvimonas micra*, and *Campylobacter jejuni* in CRC patients, while probiotics like *Bifidobacterium longum* were found to be down-regulated (Chen et al., 2022).

Moreover, Combining FIT with the abundance of specific bacteria can significantly improve the diagnostic performance of CRC. The experimental study showed that the ratio of *Fn* to *Bifidobacterium* had high sensitivity and specificity (84.6 and 92.3% respectively) in the early diagnosis of CRC. When the ratio of *Fn* to *Bifidobacterium* and the ratio of *Fn* to *Brucella* were combined for the diagnosis of CRC, the area under the curve was 0.943, indicating that the ratio of *Fn* to *Brucella* and *Bifidobacterium* could improve the specificity of early diagnosis of CRC. The combination of the two had a good diagnostic value in screening for early CRC (Guo et al., 2018). In a similar study, a comprehensive analysis of fecal samples from all participants was conducted using shotgun metagenomic sequencing, and *β*-diversity was evaluated through NMDS analysis. This investigation revealed significant differences in the bacterial community structure among the CRC, colorectal adenoma (CRA), and normal control (NC) groups. Notably, bacterial species such as *Peptostreptococcus stomatis, Fn, Parvimonas micra, Peptostreptococcus anaerobius* and *Bacteroides fragilis* were significantly elevated in CRC patients. Conversely, species such as *Coprobacter fastidosus, Eubacterium ventriosum, Roseburia interinalis and Roseburia inulivorans* were markedly reduced. In addition, moving from NC to CRA to CRC, *Leptotrichia buccalis* and *Prevotella veroralis* numbers rose incrementally, while *Lachnospiraceae bacterium 1_4_56FAA* and *Eubacterium dolichum* numbers decreased gradually (Coker et al., 2022). The relative abundance of ETBF in feces and tissues of healthy controls, CRA and CRC patients increased stepwise, and the high expression of ETBF and its BFT gene can be clearly detected in clinical specimens of CRC patients. At the same time, the occurrence of CRC induced by ETBF depends on the down-regulation of miR-149-3p. In clinical samples, the content of miR-149-3p in exosome-packaged exosomes was significantly decreased in CRC, which was negatively correlated with the abundance of ETBF in IBD and CRC patients (Cao et al., 2021).

Fecal and urinary extracellular vesicles (EVs) in patients with CRC reflect a unique intestinal microbiome that can be used as a new marker for CRC detection and prognosis. EVs derived from Alistipes were significantly increased in the urine and feces of patients with early and advanced CRC. Compared with healthy individuals, urinary and fecal EVs derived from gut microbes were significantly different in microbial composition, evenness, and diversity of CRC subjects. Compared with early CRC subjects with fecal microbiota and intestinal microbial-derived EV, the microbial composition, evenness, and diversity of late CRC subjects changed significantly (Park et al., 2021; Yoon et al., 2023).

## 4 The role of intestinal flora in the prevention and treatment of CRC

The effect of intestinal flora on CRC has been gradually elucidated. Based on these findings, therapeutic regimens that directly or indirectly regulate intestinal flora to improve the prognosis of CRC patients have emerged and have been gradually applied in clinical practice. After thoroughly discussing how microorganisms affect CRC, we will now focus on converting these findings into practical diagnostic and treatment methods.

### 4.1 Diet control

Dietary intake is a key determinant of the production of intestinal flora, short-chain fatty acids (SCFAs), and branched-chain fatty acids (BCFAs) (Bourdeau-Julien et al., 2023). Diet plays a pivotal role in modulating the intestinal microbiota, thereby influencing the likelihood of CRC development. Consuming a whole-grain diet has been shown to significantly lower the incidence of CRC (Gaesser, 2020). Dietary fiber-rich foods, including whole grains, fruits, and vegetables, serve as nourishment for probiotics, fostering their proliferation and curbing the overgrowth of detrimental bacteria. Moreover, a diet that limits high-fat, high-sugar, and processed foods contributes to the preservation of healthy intestinal flora. At the same time, the intake of red meat and processed meat should be reduced. Some components of these meat products can produce potentially pathogenic metabolites under the metabolism of intestinal flora, such as trimethylamine, trimethylamine oxide, and sulfur dioxide, which may directly lead to the occurrence of CRC. For every 100 g increase in daily intake of red meat and processed meat, the risk of CRC will increase by 12% (Vieira et al., 2017; Song et al., 2020). The biological mechanism of unhealthy dietary patterns leading to CRC may be multifaceted and is the result of the interaction of various dietary components. Therefore, a reasonable diet, a healthy diet, eat more fruits and vegetables, is an effective way to prevent CRC.

### 4.2 Use of microbial agents

Supplementation of exogenous microbial preparations (such as probiotics) to regulate intestinal microecology to prevent or treat CRC. Probiotics in the intestinal flora play a key protective role in preventing and treating human tumors. These probiotics can affect the host’s physiological and immune mechanisms, and may further play a regulatory and anti-tumor activity role (Cristofori et al., 2021). In particular, *Lactobacillus* and *Bifidobacterium* spp., the two most common probiotics, have been shown to enhance gastrointestinal function and improve the immune function of the intestinal system (Sanders et al., 2019).

*Lactobacillus* is a genus of Gram-positive bacteria characterized by its ability to exist in single, paired, or short chain arrangements. These bacteria primarily engage in anaerobic respiration and exhibit a robust capacity to metabolize carbohydrates, resulting in the production of acids. Additionally, they synthesize glucans and heteropolysaccharides and ferment carbohydrates to generate substantial quantities of lactic acid. This metabolic activity is crucial for maintaining the health of humans and higher animals. In patients with CRC, the administration of Lactobacillus strains, either as monotherapy or in conjunction with standard chemotherapeutic agents, may exert a beneficial impact on tumor development and progression. This effect is mediated through various mechanisms, including the induction of apoptosis, modulation of immune responses involving inflammatory cytokines, and alterations in the composition and characteristics of intestinal microbial communities. In addition, these strains can also affect the signal transduction pathways involved in cell migration and invasion, thereby effectively curbing tumor metastasis (Ghorbani et al., 2022).

*Bifidobacterium*, a strictly anaerobic, gram-positive, and polymorphic rod-shaped bacterium, that plays an important role in inhibiting the colonization of intestinal pathogens, maintaining the homeostasis of intestinal microorganisms, and protecting the integrity of the intestinal mucosal barrier (Gavzy et al., 2023). At the same time, *Bifidobacterium* can regulate the composition of intestinal microbiota in mice, improve intestinal barrier function, block pro-inflammatory cytokines, and inhibit the TLR4/ NF-κB pathway, thus effectively alleviating DSS-induced colitis (Chen et al., 2021; Wang et al., 2021). Relevant experimental data have shown that the metabolites secreted by *Bifidobacterium* have anti-cancer and apoptosis-inducing effects on colon cancer cell lines. It is posited that the mechanism underlying the cancer-preventive effect against CRC involves the down-regulation and up-regulation of anti-apoptotic genes and pro-apoptotic genes (Faghfoori et al., 2021).

### 4.3 Fecal microbiota transplantation (FMT)

FMT refers to the transplantation of intestinal flora from healthy donors into the intestinal tract of patients. By reconstructing the intestinal flora to treat the disease, the diversity of intestinal flora in patients can be quickly restored, thereby improving the disease status (Fong et al., 2020). A number of animal experimental data have shown that FMT can restore the composition and diversity of intestinal flora and inhibit the occurrence of colonic inflammation and tumor cell proliferation (Wang et al., 2019; Bell et al., 2022). Furthermore, research has demonstrated that FMT can significantly mitigate the adverse reactions experienced by patients with CRC during surgical procedures, chemotherapy, radiotherapy, and immunotherapy, thereby offering novel strategies to alleviate treatment-related side effects (Bokoliya et al., 2021). At the same time, FMT may also have the potential to enhance the efficacy of anti-cancer treatment and optimize the prognosis of patients. This indicates that FMT combined with other treatment methods for CRC has high clinical application value and broad development prospects.

## 5 Conclusion and prospect

Over recent years, the relationship between the intestinal microbiota and the genesis and progression of CRC has become a focal point in medical science. This microecology serves as a critical link between environmental influences and the host, with its role in CRC development garnering heightened scrutiny. The investigation into how the intestinal flora modulates the intestinal milieu to potentially foster CRC development is of significant interest. This exploration is crucial for establishing a robust theoretical framework that can inform strategies for CRC prevention.

Under healthy conditions, there is a delicate balance between the intestinal flora and the host. However, when this balance is broken, that is, the occurrence of dysbacteriosis, may trigger a series of diseases, and may even lead to the occurrence of CRC (Colella et al., 2023). Related studies have identified links between this shared gut ecological imbalance across multiple diseases and factors such as heredity, childhood and current exposures, lifestyle, and socioeconomics. Additionally, high-carbohydrate diets, smoking, and air pollutants are associated with unhealthy microbiome profiles (Gacesa et al., 2022). Intestinal flora composition changes significantly with the host’s life cycle and environment. In CRC mouse models, tumor development shows sexual dimorphism, potentially explaining gender differences in CRC rates. Male mice exhibit more pathogenic bacteria and fewer probiotics, with specific metabolites activating glycerophospholipid pathways that worsen colorectal tumors, closely tied to CRC progression (Wang et al., 2023). As an additional important factor, a high-fat diet (HFD) directly promotes the growth of colon cells, activates tumor-related genes, and weakens the intestinal barrier’s integrity by inducing an imbalance in the intestinal flora and metabolite profile (Yang et al., 2022). This variability provides an important basis for microbiome-based personalized medical strategies, including early diagnosis, treatment and health management of diseases.

At present, there are still limitations in the study of intestinal microbes in colorectal cancer. Although a number of prospective studies have confirmed the potential of intestinal flora as a diagnostic marker for CRC, the translation of these findings into clinical applications still faces multiple challenges. The pattern of microbiome dysbiosis is significantly heterogeneous among different studies and is susceptible to a combination of internal and external factors, including genetic background, geographical location, diet, and drug use. How these environmental variables lead to differences in bacterial abundance remains to be explored, and how to eliminate the interference of these differences to find universally applicable microbial markers and interventions becomes difficult. It is worth noting that recent studies have found that the differences in sequencing results between different technical solutions even exceed the natural differences between populations, posing a serious challenge to the comparability of cross-study data (Liu et al., 2023).

Investigating the link between intestinal flora and CRC represents a burgeoning area of research. This field offers novel insights into carcinogenesis mechanisms and may pave the way for innovative preventive and therapeutic approaches. The role of microbiota modulation is particularly unique in the clinical management of CRC. Through the targeted manipulation of the intestinal flora, there is potential to counteract CRC effectively. This approach could facilitate personalized therapeutic plans or serve as an adjunct to existing treatments, ultimately enhancing patient outcomes and overall quality of life. Meanwhile, in recent studies, biomarkers of CRC can be identified based on the use of Explainable Artificial Intelligence (XAI) to analyse gut microbiome data. The development of this technology opens the way for this approach to provide targeted interventions based on microbial profiles, as well as insights to fine-tune personalised diagnostic and therapeutic strategies (Novielli et al., 2024). There is also the possibility of finding new targets, similar to the MAPK pathway, for the treatment of CRC (Zhou et al., 2025).

There are complex links between the intestinal flora and human health. Nonetheless, several hurdles such as personal variations, technical difficulties, and ethical and regulatory concerns must be addressed to apply intestinal flora research findings in clinical settings. Although it has been shown that imbalances in the gut flora may be associated with the development of CRC, the exact mechanisms by which these flora directly contribute to cancer development remain to be further elucidated. There are still doubts about the definite mechanism of the direct effect of intestinal flora on CRC, only by analyzing the activation of multiple signaling pathways and the up-regulation of some inflammatory cytokines, leading to the transformation of normal intestinal epithelial cells into malignant cells, to explain the connection (Wong and Yu, 2023). Therefore, before using intestinal flora to achieve beneficial therapeutic effects on CRC, more animal and clinical experimental data are needed to further improve the mechanism of intestinal flora on CRC. As research delves deeper into the influences of age and environment on the microbiota, it is anticipated that forthcoming comprehensive studies will enhance the translation of microbiome research into clinical practice. This advancement is poised to become a crucial element in cancer diagnosis, prevention, and treatment, thereby paving the way for more effective personalized medicine strategies (Nouri et al., 2022).
